# Altered Neuroinflammatory Transcriptomic Profile in the Hippocampal Dentate Gyrus Three Weeks After Lateral Fluid Percussion Injury in Rats

**DOI:** 10.3390/ijms26189140

**Published:** 2025-09-19

**Authors:** Anthony J. DeSana, Yara Alfawares, Roshni Khatri, Tracy M. Hopkins, Faith V. Best, Jennifer L. McGuire, Laura B. Ngwenya

**Affiliations:** 1 Department of Neurosurgery, University of Cincinnati College of Medicine, Cincinnati, OH 45267, USA; desanaaj@ucmail.uc.edu (A.J.D.); alfawaya@mail.uc.edu (Y.A.); morttl@ucmail.uc.edu (T.M.H.);; 2 Department of Neurology and Rehabilitation Medicine, University of Cincinnati College of Medicine, Cincinnati, OH 45267, USA

**Keywords:** traumatic brain injury, dentate gyrus, transcriptomics

## Abstract

Traumatic brain injury (TBI) is a major source of disability worldwide, with cognitive and memory deficits being pervasive after injury. The hippocampus, a major structure involved in learning and memory, is particularly vulnerable to TBI, and cellular dysfunction within the hippocampal dentate gyrus is believed to be a major contributor to cognitive deficits after TBI. However, there is little known about the transcriptomic changes occurring directly within the dentate gyrus at subacute-to-chronic timepoints after TBI. To address this, we performed bulk RNA sequencing and single-nucleus RNA sequencing of the isolated dentate gyrus three weeks after lateral fluid percussion injury in male rats. We report here that there is evidence of an ongoing neuroinflammatory response marked by increased neuroinflammatory genes that implicate various neuroinflammatory pathways that are associated with a subset of microglia and astrocyte populations.

## 1. Introduction

Traumatic brain injury (TBI) is estimated to affect sixty-nine million people annually, with long-term implications regarding cognitive abilities after injury [1,2]. Clinically, cognitive and memory impairments are commonly reported after TBI [3,4,5] and are recapitulated in pre-clinical models of TBI [6,7,8]. Cognitive dysfunction is associated with TBI-induced changes to the hippocampus [9], and the hippocampal dentate gyrus is particularly vulnerable to TBI [10,11,12]. Given the dentate gyrus’s role in cognition, learning, and memory (reviewed in [13]), dentate gyrus dysfunction likely plays an important role in the cognitive and memory deficits observed after TBI. Early after TBI, there is a loss of hippocampal neural progenitor cells within the subgranular zone of the dentate gyrus, followed by increased neurogenesis from 24 h to 7 days post-injury [10,14,15,16], with reduced astrogliogenesis [15].

Under healthy conditions, glial cells play important roles within the dentate gyrus. Retention and integration of new-born neurons are mediated by local microglia and astrocytes [17,18]. Microglia are necessary for survival of neuronal stem cells [19], and interplay between microglia and astrocytes facilitates synaptic pruning [20]. In the context of TBI, astrocytes and microglia within the dentate gyrus demonstrate a robust response to TBI associated with the neuroinflammatory response to injury [21,22,23]. Neuroinflammatory changes are well documented after brain injury and are believed to be a key driver of long-term cognitive dysfunction (reviewed in [24]). Hippocampal transcriptional changes associated with an ongoing immune response occur at early, subacute, and chronic timepoints post-injury [25,26], and transcriptional changes in animals have been correlated with long-term TBI-associated neurodegenerative sequelae [27].

Researchers have long recognized the importance of the dentate gyrus after TBI, and, in the past decade, a plethora of publications have examined the TBI-induced transcriptional changes exhibited in animal brains, and specifically the hippocampus [28,29,30,31,32]. However, the majority of such studies focus on the acute phase after injury, mostly obtaining data within 24 h to 7 days post-injury. Importantly, little is known about changes in gene expression at timepoints after 2 weeks post-injury, especially in the isolated dentate gyrus [15,33].

Given the enduring effects of TBI on cognitive function, we aimed to utilize both bulk and single-nucleus RNA sequencing techniques to examine the transcriptional changes in the isolated dentate gyrus at three weeks after TBI. To our knowledge, this is the first single-nucleus study to investigate the whole tissue dentate gyrus specifically after rat TBI, providing a greater level of regional resolution within the injured hippocampus. Briefly, our data suggest altered gene expression associated with an ongoing neuroinflammatory response within specific populations of microglia and astrocytes at three weeks post-TBI.

## 2. Results

**Figure 1:**
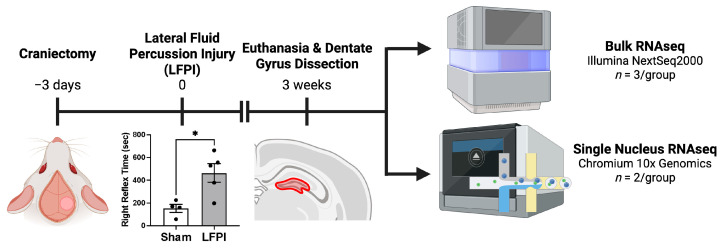
Experimental timeline: Rats underwent a craniectomy three days prior to TBI induced by lateral fluid percussion injury (LFPI) or sham injury. TBI rats had significantly increased righting reflex time compared to sham animals (mean ± standard error; * corresponds with a *p* < 0.05; *p* = 0.0146). Three weeks post-injury, rats were euthanized, and the ipsilateral hippocampal dentate gyrus was dissected out and processed for bulk RNA sequencing (NextSeq2000; Illumina, Inc., San Diego, CA, USA) or single-nucleus RNA sequencing (Chromium 10x Genomics; 10x Genomics, Inc., Pleasanton, CA, USA). Created in BioRender. DeSana, A. (2025) https://BioRender.com/eyfapfv (accessed on 30 July 2025).

Male Sprague-Dawley rats underwent a craniectomy, and, three days afterward, underwent a lateral fluid percussion injury (LFPI) to induce TBI. As an additional confirmation of injury, spontaneous righting reflex time (RRT) was assessed. Brain-injured rats had significantly longer RRT compared to sham when compared with a two-tailed unpaired *t*-test (*p* = 0.0146, *t* = 3.221, df = 7; Figure 1). The rats were euthanized three weeks after injury, and the dentate gyrus was dissected out and processed for bulk RNA-seq (*n*_total_ = 6) or single-nucleus RNA-seq (snRNA-seq; *n*_total_ = 4; Figure 1).

### 2.1. Bulk RNA Sequencing Reveals a Neuroinflammatory Response in the Dentate Gyrus Three Weeks After LFPI

**Figure 2:**
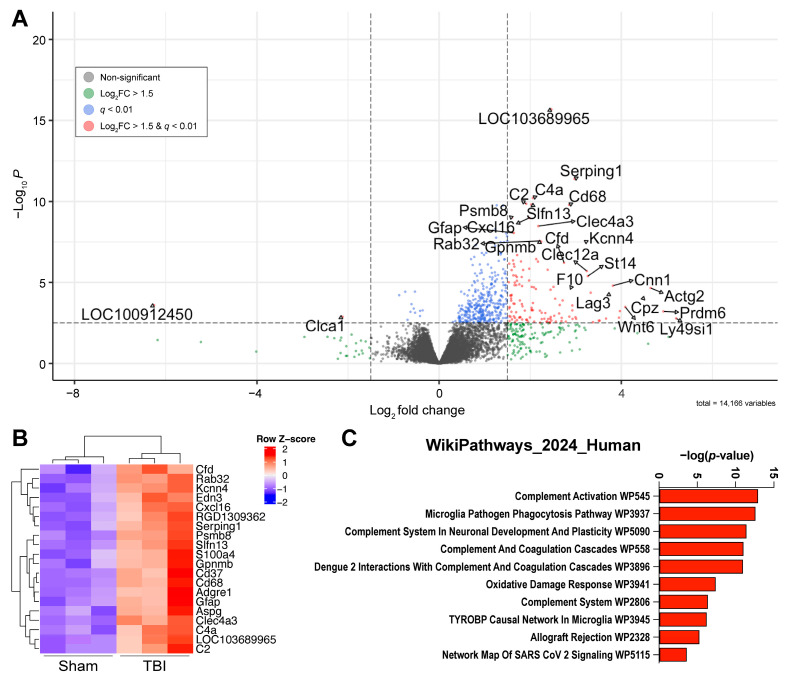
Differentially expressed genes suggest upregulation of genes involved in neuroinflammation and complement activation: following TBI, 126 genes were upregulated in TBI rats, with 2 genes downregulated compared to sham injured rats. (**A**) Volcano plot representing differentially expressed genes (*q* < 0.01 and Log2FC > 1.5), where each dot represents an individual gene. Genes with a Log2FC > 0 represent an increase in TBI rats compared to sham rats, and Log2FC < 0 represents a decrease in TBI compared to sham. (**B**) Hierarchical clustering with complete linkage shows a separation in gene expression between sham and TBI rats, with various genes involved in neuroinflammation upregulated with injury. (**C**) Pathway analysis mapped to the ‘WikiPathways 2024 Human’ database further suggests neuroinflammatory activation in the dentate gyrus at 3 weeks post-TBI, and several of the implicated pathways involve complement activation.

As a result of our bulk RNA sequencing, a total of fourteen-thousand-three-hundred-sixty-eight genes were identified, with four-hundred-seventy-six having a *q*-value < 0.01 (Benjamini and Hochberg). Of these, one-hundred-twenty-eight genes were differentially expressed (*q* < 0.01; Log2FC > 1.5). Of these differentially expressed genes (DEGs), one-hundred-twenty-six were upregulated in TBI and two were downregulated (Figure 2A). Hierarchical clustering with complete linkage shows that injured and sham rats cluster separately, and that rats within the same injury group are similar to each other (Figure 2B). Notably, genes associated with inflammatory processes were upregulated in brain-injured rats, with the top three DEGs based on *p*-value being *LOC103689965* (*p* = 3.21 × 10^−13^, Log2FC = 2.47), *Serping1* (serpin family G member 1; *q* = 3.11 × 10^−09^, Log2FC = 2.97), and *C4a* (*q* = 3.10× 10^−08^, Log2FC = 2.06), which encode for the proteins ‘Complement C4-like’, ‘C1 inhibitor’, and the acidic form of ‘Complement Factor 4’. The two downregulated genes, *LOC10091245* (*q* = 0.00178, Log2FC = −6.25) and *Clca1* (*q* = 0.005616, Log2FC = −2.12), encode for SMCO3 and CLCA1 (Chloride Channel Accessory 1), respectively. While SMCO3’s function has not been well characterized, *Clca1* is of potential interest as it has been implicated in glutamate-induced cell death within the hippocampus [34]. Under ischemic conditions, extrasynaptic *N*-methyl-D-aspartate (NMDA) activation increases *Clca1* gene expression within hours and has been shown to induce cell death in vivo [35]. The implications of the downregulation of *Clca1* gene expression three weeks after LFPI remain to be determined. 

A pathway analysis through Enrichr using the “WikiPathways 2024 Human” dataset implicated multiple complement-related pathways. Of the top 10 pathways identified by Enrichr, half are directly associated with complement signaling (Table 1). Additionally, multiple complement genes are implicated as part of the “Microglia Pathogen Phagocytosis pathway”. These findings suggest an ongoing inflammatory response within the dentate gyrus at three weeks after injury.

### 2.2. A Subset of Dentate Gyrus Microglia Express Genes Associated with an Activated Phenotype

Because bulk RNA sequencing does not provide information on the cellular source of observed transcriptional changes, we chose to perform additional single-nucleus RNA sequencing experiments to identify the sources of neuroinflammation in the dentate gyrus. Due to the prominent change in inflammatory-related genes observed in our bulk RNA sequencing, we chose to focus our single-nucleus RNA sequencing data analysis on microglia and astrocytes as these were the most likely source of the observed changes in complement-related genes [36,37]. Under pathological conditions, both microglia and astrocytes have been implicated in clearing debris [38], synaptic remodeling [39,40], and progressive neurodegeneration (reviewed in [41,42]).

**Figure 3:**
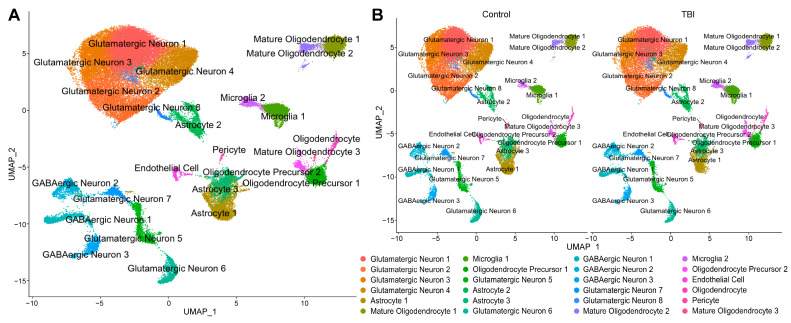
Single-nucleus RNA sequencing clustering: (**A**) UMAP clustering of nuclei shows 24 distinct clusters that were assigned to glutamatergic neurons, GABAergic neurons, astrocytes, microglia, oligodendrocyte precursor cells, oligodendrocytes, mature oligodendrocytes, endothelial cells, and pericytes. (**B**) When the UMAP was split by injury condition, some differences in intercluster relationships are appreciable.

Prior to quality control and filtering (detailed below), 55,863 nuclei were present in our samples, and, afterwards, 55,123 nuclei were utilized for analysis. A Uniform Manifold Approximation and Projection (UMAP) graph was generated using Seurat, and 24 distinct clusters were identified (Figure 3A). Using the online tool, “Annotation of Cell Types” (ACT) [43], clusters were labeled as astrocytes, microglia, oligodendrocytes, oligodendrocyte precursor cells, mature oligodendrocytes, pericytes, endothelial cells, GABAergic neurons, and glutamatergic neurons. Two clusters were identified as microglia, and three clusters were identified as astrocytes. When the UMAP was split by injury condition (Figure 3B), there were some appreciable differences in the number and distribution of nuclei within the microglial and astrocyte clusters (Table A1). The TBI animals had a greater number of nuclei associated with both microglial clusters (Microglia 1 and Microglia 2), while the astrocyte nuclei were lower in TBI animals in Astrocyte 1 and 3 but were higher in TBI animals in Astrocyte 2 when compared to sham.

**Figure 4:**
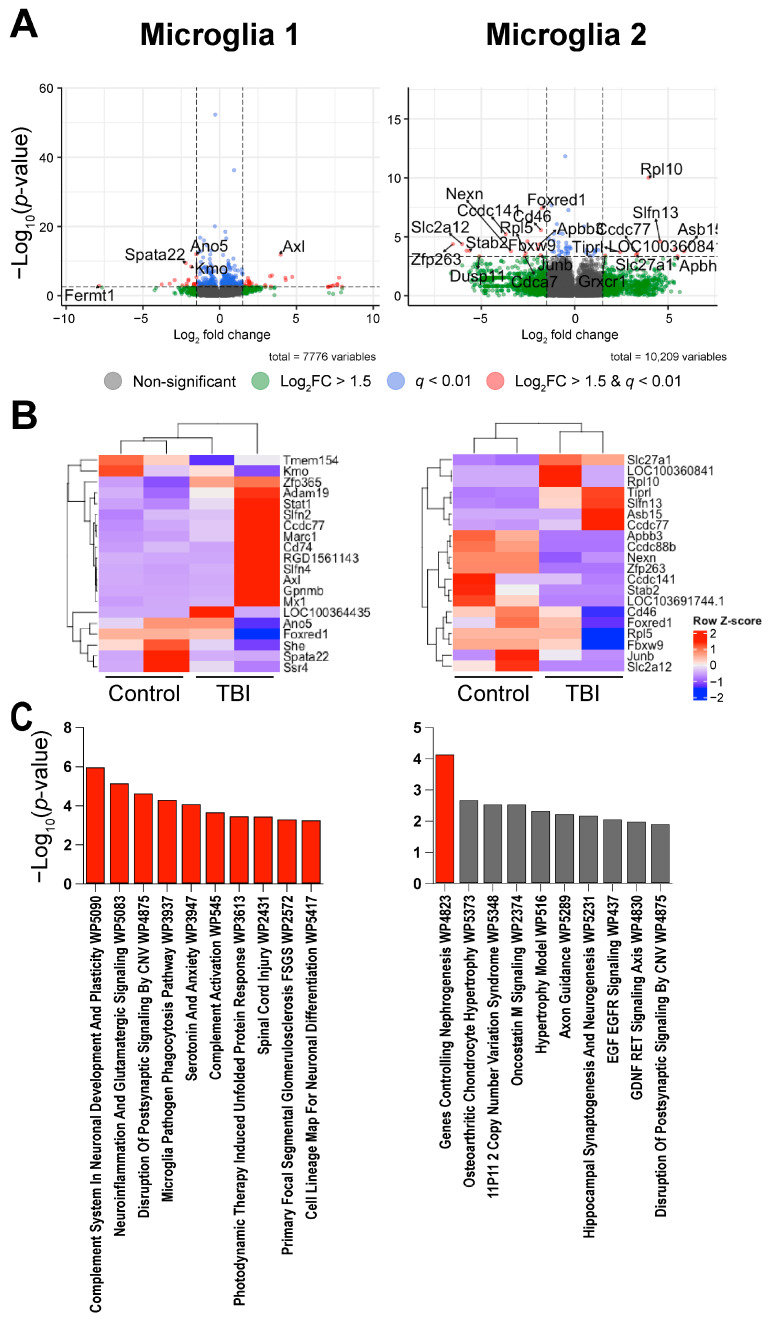
Single-nucleus RNA sequencing suggests microglial involvement in complement signaling: (**A**) differential expression analysis within each microglial cluster revealed significantly up- and downregulated genes (*q* < 0.01 and Log2FC > 1.5) between TBI and control animals, with (**B**) hierarchical clustering with complete linkage showing a clear separation between control and TBI rats in the Microglia 2 cluster, with less consistent separation between groups in the Microglia 1 cluster. (**C**) Pathway analysis revealed that the nuclei from Microglia 1 likely represent a pro-inflammatory microglial population with evidence of complement signaling, while Microglia 2 likely represents a homeostatic microglial population. Red bars represent significant findings by Fisher exact test with Benjamini and Hochberg correction: q < 0.05, while gray bars represent non-significant findings.

Differential expression analysis between the control and TBI rats revealed that fifty-four genes were differentially expressed in Microglia 1 (thirty-four upregulated and twenty downregulated in TBI; Table 2) and twenty-four genes in Microglia 2 (nine upregulated and fifteen downregulated in TBI). The top five DEGs based on *p*-value were *Ano5*, *Axl*, *Spata22*, *Kmo*, and *Slfn4* for Microglia 1 and *Rpl10*, *Foxred1*, *Cd46*, *Ccdc141*, and *Ccdc88b* for Microglia 2. A hierarchical clustering analysis by complete linkage clustering (Figure 4) did not show clear clustering between the injury and control groups for Microglia 1 but did cluster by injury condition for Microglia 2. Similar to what was observed in our bulk signaling, pathway analysis shows that the DEGs associated with Microglia 1 are enriched for complement signaling. The most significant pathway (Table 3) identified was “Complement System in Neuronal Development and Plasticity” (*p* = 1.03 × 10^−6^). However, when looking at the complement-associated DEGs, it is evident that several complement genes are decreased in our TBI animals compared to control animals, in opposition to what was observed in our bulk RNA sequencing. This included a reduction in the *C1qa*, *C1qb*, and *C1qc* components of the C1q subunit that helps form the C1 complex and is associated with classical complement signaling [44]. The second most significant is “Neuroinflammation and Glutamatergic Signaling” (*p* = 6.59 × 10^−6^). Only one pathway was found to be significant in the Microglia 2 cluster after multiple testing corrections, “Genes controlling Nephrogenesis” (*p* = 6.85 × 10^−5^).

Despite the discrepancy between our bulk and single-nucleus data, several inflammatory-associated genes were upregulated in Microglia 1, suggesting that there is still an inflammatory response present within the dentate gyrus at three weeks post-TBI. These genes include three genes that are associated with reactive microglia: *Axl* (Log2FC = 3.97, *p* = 1.41 × 10^−12^), *Cd74* (Log2FC = 1.84, *p* = 7.92 × 10^−6^), and *Stat1* (Log2FC = 1.77, *p* = 7.10 × 10^−5^) [45,46,47].

**Figure 5:**
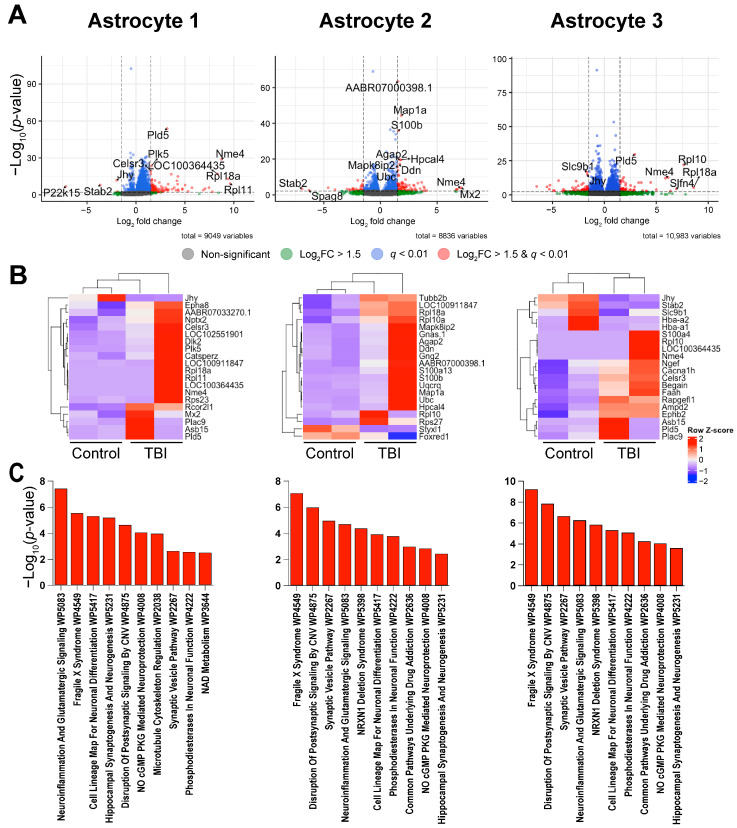
Changes in astrocyte gene expression three weeks after TBI: (**A**) differential expression analysis within each cluster revealed significantly up- and downregulated genes (*q* < 0.01 and Log2FC > 1.5) within the three astrocyte clusters between control and TBI animals. (**B**) Hierarchical clustering analysis showed inconsistent separation between the Astrocyte 1 and Astrocyte 2 clusters but clear separation in the Astrocyte 3 cluster. (**C**) Pathway analysis suggests that all three astrocyte clusters show evidence of increased neuroinflammation and altered signaling. Red bars represent significant findings by Fisher exact test with Benjamini and Hochberg correction: *q* < 0.05.

For the astrocyte clusters (Figure 5), one-hundred-ten DEGs were identified in Astrocyte 1 (one-hundred-five upregulated and five downregulated; Table 4), one-hundred-four DEGs were identified in Astrocyte 2 (seventy-six upregulated and twenty-eight downregulated), and one-hundred-thirty-five DEGs were identified in Astrocyte 3 (sixty upregulated and seventy-five downregulated). A hierarchical clustering analysis using complete linkage clustering (Figure 5B) did not show clear clustering between injury groups in the clusters for Astrocyte 1 and Astrocyte 2 but did cluster by injury condition in Astrocyte 3. A pathway analysis for all three astrocyte clusters suggests changes in genes associated with neuroinflammation and altered synaptic signaling (Table 5).

There was a disproportionate amount of upregulated DEGs in our bulk RNA sequencing data compared to most microglial and astrocyte clusters in our single-nucleus dataset. This skew towards upregulated genes in our bulk data may be due to our exclusion of neuronal cell populations from our complete analysis. As post-mitotic cells, neurons have high transcriptional activity compared to other cell types in the brain [48]. This may also reflect changes in the abundance of specific mRNA transcripts outside the nucleus due to post-translational modifications that can influence mRNA abundance and longevity [49]. Alternatively, it may be due to technical differences between bulk and single-nucleus RNA sequencing. Bulk sequencing averages out the expression of genes across cell types, and as such the differential regulation of the same gene between different cell types can eliminate changes that are appreciable at single-cell resolutions. Additionally, single-nucleus RNA sequencing generally captures a smaller subset of available RNA because it does not include RNAs found outside of the nucleus [50].

Taken together, there is evidence for an overall increase in neuroinflammation-related genes despite lacking complete accordance on the pathways that are increased at three weeks after TBI. Ultimately, the differences in these datasets underscore the complementary nature of bulk RNA sequencing and single-nucleus sequencing technologies in untangling the complex neuroinflammatory response to TBI.

## 3. Discussion

Given the high incidence of TBI and its enduring implications on cognitive function, our aim was to assess the transcriptional changes that cells in the dentate gyrus of the hippocampus undergo at three weeks after TBI. Briefly, our data showed a significant upregulation in the transcription for components of the complement system in the bulk-sequenced specimens. Due to the overrepresentation of inflammatory pathways in our bulk sequencing, we chose to focus on microglial and astrocyte clusters when analyzing our single-nucleus RNA-seq dataset. The microglia DEGs were enriched for pathways associated with phagocytosis, while the astrocytes exhibited enrichment in pathways for neuroinflammation and glutamatergic signaling as well as hippocampal synaptogenesis and neurogenesis.

Our bulk RNA sequencing findings are consistent with a number of studies in clinical and animal models that demonstrated a role for complement activation after TBI. Under healthy conditions, the complement system is a component of the innate immune system, which comprises a family of proteins that activate one another in an amplification cascade, usually culminating in inflammatory signaling, opsonization, or removal of foreign bodies or cellular debris [51]. The complement system performs these roles in the brain, protecting it from infection [52] and removing fragments of apoptotic cells as well as aggregated proteins [53,54]. More interestingly, it contributes to synaptic pruning by way of astrocytes, which express the complement proteins, opsonizing specific synapses for phagocytosis by microglia [36,37,55]. Additionally, it plays a role in neurogenesis, with some mediators (C3 and C5) encouraging maturation and migration and others (C3d and CR2) inhibiting them [56,57,58].

In contrast, the complement system takes on a more pro-inflammatory role in the brain after trauma, contributing to secondary injury. Bellander et al. found increased presence of complement components C1q, C3b, C3d, and the membrane attack complex (MAC) composed of C5b-9 in the penumbra around the contused brain tissue [59], whereas Kossmann et al. found increased levels of C3 and factor B (fB) in the CSF of TBI patients [60]. Moreover, numerous studies in animal models have shown supporting findings by manipulating the complement system after TBI. Kaczorowski et al. inhibited the system with the use of soluble CR1 (sCR1) and found a decrease in neutrophil infiltration into brain tissue [61]. Rancan et al. showed that transgenic mice expressing a soluble complement inhibitor (sCrry) experienced less neurologic impairment after TBI compared to wild-type mice [62]. Leinhase et al. built on this by injecting brain-injured mice with recombinant sCrry, finding that inhibiting the complement system in this way allowed for decreased tissue destruction in the hippocampus and better neurologic function [63], showing great promise for this system as a potential therapeutic target after TBI.

Nevertheless, with the rise of sequencing information technologies in the recent past, there is great utility in examining these system dynamics as they occur without experimental intervention, leaving room to uncover their molecular and cellular interactions more broadly. This is of particular interest as the complement system has been shown to effect change by interacting with astrocytic and microglial cell populations [36,37,54,55,64]. Indeed, transcriptomic studies, focusing mostly on the acute phase after TBI, have found increases in astrocytic and microglial cell populations along with upregulation of inflammatory pathways, including chemokine and interleukin signaling [28,65]. A similar pattern of activation and inflammatory function was detected by Arneson et al., who also interestingly uncovered an increase in calcium/calmodulin signaling by astrocytes, a finding mirrored by our data in which all three astrocyte groups showed upregulation of the *Camk2a* and *Camk2b* genes [31]. Further linking astrocytic and microglial interactions with complement activation, Zheng et al. found communication between microglia and endothelial cells via components of the complement system (C1qa-Cd93) [30]. Other studies found significant amplification of transcribed genes that were flagging pathway terms, including “complement and coagulation cascades” and “innate immunity”, in their analyses [64,66]. One of note even identified this upregulation of complement at a timepoint closer to our own, 14 days after injury, and yet another at much later timepoints up to 2 years after injury [65,67].

Interestingly, while the list of DEGs from our Microglia 1 single-nucleus cluster was significantly enriched for complement-related genes, several of the associated genes were downregulated compared to our control animals. Specifically, genes associated with the components of the C1q subunit of the C1 complex were downregulated, suggesting that the classical complement pathway was reduced in Microglia 1 nuclei. At one day and two weeks following LFPI, Catta-Preta et al. assessed the transcriptional changes within the rat hippocampus and found that a subset of genes associated with inflammatory pathways were upregulated at both timepoints, while others had a delayed upregulation [26]. In that study, several genes relating to complement signaling were persistently upregulated at both timepoints, while other complement genes were associated with delayed increase, including *C4a*, which we observed in our bulk sequencing but not our single-nucleus RNA sequencing. This study utilized bulk RNA sequencing, making it most comparable to our bulk RNA sequencing dataset and suggesting that complement signaling may be increased persistently up to three weeks post-injury.

Our results show the prevalence of complement activation in our bulk-sequenced data and are consistent with the literature. Our exploration of microglial and astrocytic activation in the single-nucleus-sequenced data provide further evidence of neuroinflammation, but either shows a downregulation or lacks many of the genes associated with complement activation that were observed in our bulk RNA sequencing. This discrepancy may be due to the differences between the two sequencing technologies [68,69] or typical localization of mRNA transcripts, but it is also possible that the observed changes in complement could be attributed to an unexpected cell type.

Delving into the astrocytic nuclei changes shows significant enrichment in pathways such as “neuroinflammation and glutamatergic signaling” but also in “hippocampal synaptogenesis and neurogenesis” by way of upregulation of genes including *Camk2a*, *Camk2b*, *Nrxn1*, and *Ptpn6*. This becomes more intriguing in the context of some transcriptomic studies after TBI, one of which found increased activity of the “innate immunity” pathways but also in those for “axon guidance”, and another found *Nup62* responsible for increasing cell division [29,32]. Additionally, transcriptional changes occurring in the chronic setting after TBI at 30 and 90 days post-injury, as noted by Makinde et al., were associated with pathways for “synaptic plasticity” and “regulation of long-term synaptic potentiation” [70]. Indeed, investigating some of our implicated genes shows that numerous studies have implicated *Camk2a, Camk2b,* and *Nrxn1* in neurogenesis and neuroplasticity through neuritic branching, pruning, and assembly [71,72,73,74,75]. Further, these astrocytic changes could be linked to complement upregulation through the overexpression of *Ptpn6* in our data, which was found to be linked to *C1s* [76], and to neuroplasticity with the overexpression of the closely related PTPN5 [70].

In our Astrocyte 2 cluster, the genes *Map1a* and *Mapk8ip2* were upregulated in the TBI animals compared to sham. These genes are important for microtubule maintenance [77,78]. However, microtubule changes in astrocytes remain relatively poorly understood [79]. In TBI-associated neurodegenerative pathologies, microtubule disturbances often occur with tau-related changes in neurons, but glial tau pathology has been observed in hippocampal astrocytes [80,81]. Tau-related changes can happen within one day after TBI in tauopathy mouse models [82], and accumulated tau has been shown to be a potent immune target and contribute to synapse loss [83].

We noted in our hierarchical clustering of Microglia 1 (Figure 4B) and Astrocytes 1 and 2 (Figure 5A,B) that the TBI animals did not cluster together with complete linkage clustering. This may be due to outliers present within our datasets as complete linkage clustering utilizes the maximum dissimilarity value between clusters and therefore is vulnerable to outliers [84]. However, it could also be due to variability in TBI severity.

We recognize that there are limitations to the present study. Notably, our results were obtained from a small group of animals. Only male rats were utilized, so the generalizability to female rats may be limited. Additionally, for our single-nucleus RNA sequencing data, we only focused on changes to microglia and astrocytes due to the prominent change in inflammatory-related genes observed in our bulk RNA sequencing data. While we chose to focus on the two microglial populations that we identified, it is necessary to point out that single-nucleus RNA sequencing may lack the sensitivity to observe all the microglial transcriptional states relevant to TBI-induced neuroinflammation [85]. There are likely important changes occurring in the other cell populations that we have identified, and differentially expressed genes from all clusters can be found in the Supplementary Materials. Neuronal populations can likely be further localized within the dentate gyrus [86], but this was outside the scope of our study. Furthermore, there was a disproportionate amount of upregulated DEGs in our bulk RNA sequencing data compared to most microglial and astrocyte clusters in our single-nucleus dataset. This skew towards upregulated genes in our bulk data may be due to our exclusion of neuronal cell populations from our complete analysis. As post-mitotic cells, neurons have high transcriptional activity compared to other cell types in the brain [48]. This may also reflect changes in the abundance of specific mRNA transcripts outside the nucleus due to post-translational modifications that can influence mRNA abundance and longevity [49]. Alternatively, it may be due to technical differences between bulk and single-nucleus RNA sequencing. Bulk sequencing averages out the expression of genes across cell types, and as such differential regulation of the same gene between different cell types can eliminate changes that are appreciable at single-cell resolutions. Additionally, single-nucleus RNA sequencing generally captures a smaller subset of available RNA because it does not include RNAs found outside of the nucleus [50]. The dataset is publicly available for further investigation at the Gene Expression Omnibus (GSE307917), and the differentially expressed genes from this study are found in the Supplementary Materials.

To our knowledge, only a single other study has assessed changes to the isolated dentate gyrus after TBI. Bielefeld et al. assessed nestin-positive cells that were isolated from the dentate gyrus 15 days post-injury by flow cytometry followed by single-cell RNA sequencing, with a primary focus on neuronal stem cell differentiation into astrocytes and neurons in male mice [15]. This study found that TBI switched neural stem cell differentiation to favor neurogenesis over astrogliogenesis, in opposition to what occurs in the healthy brain [15]. Our study utilized whole tissue from the isolated dentate gyrus without prior flow sorting. Despite our recognized shortcomings, we believe that our findings are congruent with other studies that used non-sequencing techniques, and that our study adds further evidence for prolonged neuroinflammatory changes within the dentate gyrus after TBI.

In conclusion, the aim of our study was to assess the transcriptional changes that occur in the dentate gyrus at three weeks after injury both at the global and cellular levels. Our data suggests evidence of increased neuroinflammatory response at three weeks after TBI, but our findings differed based on the sequencing technology utilized. Generally, our findings are consistent with those of other transcriptomic studies that identified roles for complement as well as other pathways for both neuroinflammation and neurogenesis. More work is warranted to further elucidate whether these pathways contribute to a detrimental or protective neuroinflammatory response.

## 4. Materials and Methods

### 4.1. Animals and Experimental Design

Male Sprague-Dawley rats (248–476 g at injury, approx. 8–15 weeks old) were obtained from Envigo (Indianapolis, IN, USA). Rats were allowed to acclimate within the University of Cincinnati animal vivarium for at least one week prior to any procedures. Vivarium temperature was maintained at a constant temperature, and animals were initially housed in pairs on a 14/10 h light/dark cycle in filter top cages with food and water available ad libitum. Following surgical procedures (detailed below), rats were singly housed with enrichment until euthanasia. As described in more detail below, rats received TBI, were euthanized three weeks after injury, and the dentate gyrus was dissected out and processed for bulk RNA-seq (*n*_Sham_ = 3, *n*_TBI_ = 3) or single-nucleus RNA-seq (*n*_Control_ = 2, *n*_TBI_ = 2). For the single-nucleus study, one sham and one naïve animal were grouped into a single control group.

All animal experiments were approved by the University of Cincinnati’s Institutional Animal Care and Use Committee, and all experiments conformed with the National Institute of Health ‘Guide for Care and Use of Laboratory Animals’ [87].

### 4.2. Lateral Fluid Percussion Injury Model

TBI was induced using a LFPI model, as previously described [88]. In brief, animals were anesthetized using isoflurane (4% induction, 2–3% maintenance) and a 4 mm diameter craniectomy was made with a trephine hand-drill centered over the right parietal cortex at 4 mm lateral to midline and 2.5 mm caudal to bregma. A Luer-lock hub was affixed to the skull using cyanoacrylate adhesive (Super Glue, Loctite, Rocky Hill, CT, USA) to form a water-tight seal. A set screw was placed on the left frontal bone, and dental cement was used to further adhere the hub to the skull. The hub was filled with sterile saline and a Luer-lock cap was screwed on.

Animals received a single dose of extended-release buprenorphine (ZooPharm, Fort Collins, CO, USA) via subcutaneous injection for analgesic. Three days after the initial craniectomy, rats were anesthetized with isoflurane (4% for 5 min) and attached to the Luer-lock end of the LFPI device. Animals were checked for minimal consciousness using a toe-pinch, and, upon first retraction in response to toe pinch, a moderate TBI was induced by delivering a fluid pulse to the intact dura (2.14 atm; stdev = 0.11). Following injury, animals were removed from the device and spontaneous RRT was recorded as an additional confirmation of injury severity. After righting, animals were immediately re-anesthetized using 4% isoflurane and the Luer-lock hub was removed, and their incision was closed. Sham animals underwent all surgical procedures except for fluid pressure pulse delivery. Naïve animals did not undergo any surgical procedures.

### 4.3. Euthanasia and Tissue Acquisition

Three weeks following TBI, animals were immobilized using DecapiCone (Braintree Scientific, Braintree, MA, USA) and euthanized by rapid decapitation using a guillotine. The whole brain was dissected out, and the hippocampal dentate gyrus was isolated as described previously [89]. In brief, the brain was removed and placed into ice-cold 1x phosphate-buffered saline (PBS). Under a dissection microscope, brains were hemisectioned at the sagittal suture, and the medial side of the hippocampus was exposed to visualize the dentate gyrus. A 27G needle tip was then used to isolate the ipsilateral dentate gyrus. Tissue was immediately flash frozen and stored at −80 °C until processing for bulk (*n* = 3/group) or single-nucleus (*n* = 2/group) RNA sequencing.

### 4.4. Bulk RNA-Seq Sample Preparation and Analysis

The isolated dentate gyrus was placed in RNAlater-Ice (Ambion, Austin, TX, USA; cat# AM7030) and stored at −20 °C until sequencing by the University of Cincinnati Genomics Epigenomics and Sequencing core for extraction, processing, and sequencing. RNA-seq library preparation was conducted using NEBNext Ultra II Directional RNA Library Prep Kit (New England Biolabs, Ipswich, MA, USA; cat#E7760), as per manufacturer instructions, and polyA RNA sequencing was conducted using an Illumina NextSeq 2000 (Illumina, Inc., San Diego, CA, USA) with paired end reads (2 × 61bp). Adapter sequences (AGATCGGAAGAGCACACGTC; AdapterRead2: AGATCGGAAGAGCGTCGTGT) were trimmed from reads and demultiplexed prior to exporting FASTQ files from Illumina BaseSpace (Illumina, Inc., San Diego, CA, USA).

**Figure A1:**
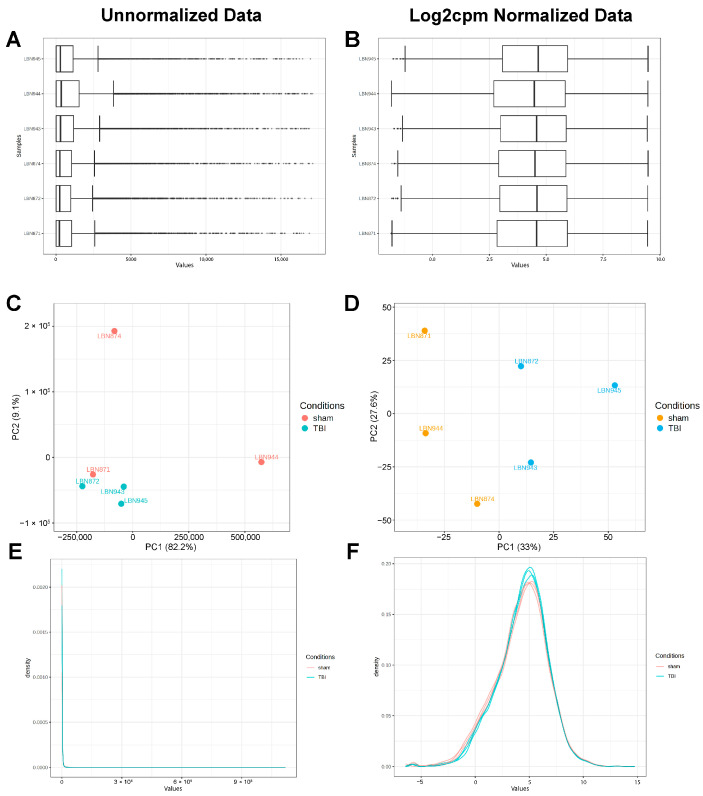
Normalization of bulk RNA sequencing data: Data normalization by Log2 counts per million: within ExpressAnalyst, data was normalized using Log2 counts per million (Log2cpm). (**A**) Distribution of read counts prior to normalization and (**B**) after Log2cpm transformation. (**C**) PCA plot showing contributions of PCs prior to normalization and (**D**) after normalization. (**E**) Plot of read density prior to normalization and (**F**) after Log2cpm transformation.

Using the ExpressAnalyst.ca platform [90] (within a Docker container on Mac OS: Apple M3 Pro, 36GB memory), FASTQ paired-end reads were uploaded and processed and mapped to the reference for Rattus norvegicus (GCF_000001895) using Kallisto under the default settings (min # reads per gene = 20). After mapping, the RNA count table was uploaded to ExpressAnalyst’s web-browser-based tools for differential expression analysis. Data was first filtered and normalized. Unannotated features were filtered out. Across the 6 samples, 31,715 features were initially identified by ExpressAnalyst, and 87% were successfully annotated (27,678 features). On average, samples had 3.02 × 10^7^ counts prior to filtering and normalization. Low-abundance samples were excluded using the sum method with a low abundance filter threshold of 4 (default). Features with a variance percentile rank lower than 15 were excluded so that only genes expressed across conditions were included. Samples were normalized by Log2 counts per million (logCPM) transformation for visualizations only (Figure A1).

Differential expression analysis was conducted using DESeq2 within ExpressAnalyst, comparing injury condition (sham vs. TBI) as the primary factor with no secondary factor. Unnormalized counts were input into DESeq2 as normalization is integrated into the DESeq2 workflow. DEGs with a *p* < 0.05 were adjusted for multiple testing within GraphPad Prism (version 10.0.3; GraphPad Software Inc., Boston, MA, USA) based on FDR (*Q* = 1%; Benjamini and Hochberg), and a *q* < 0.05 was considered significant. Genes with an absolute Log2FC ≥ 1.5 were considered to be significantly differentially expressed. DEGs were plotted as volcano plots using the EnahancedVolcano (v1.22.0) package within R (version 4.4.1) [91]. To generate heatmaps, z-scores were calculated for the top 20 DEGs ranked by *p*-value and were plotted using the ComplexHeatmap (v2.20.0) package [92]. Hierarchical clustering within heatmaps was conducted utilizing ComplexHeatmap’s default complete linkage method.

### 4.5. Single-Nucleus RNA-Seq Sample Preparation and Analysis

**Figure A2:**
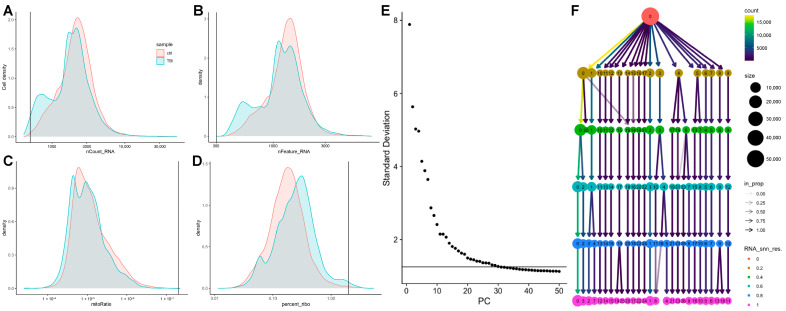
Quality control for single-nucleus RNA sequencing: Prior to normalization, data was assessed for several common quality control metrics. The traditional cutoffs were utilized: (**A**) the distribution of UMI (nCount_RNA) for control and TBI animals. The vertical line represents the traditional cutoff point (500) used to exclude nuclei that have not been sequenced deeply enough. (**B**) The distribution of genes (nFeature_RNA) with the vertical line representing the cutoff of 300 features for the exclusion of nuclei. (**C**) Distribution of the ratio of mitochondrial genes expressed to non-mitochondrial genes detected per nucleus, with the vertical line representing the cutoff for poor-quality samples (mitoRatio > 0.2). (**D**) Distribution of the ratio of ribosomal genes, with the vertical line representing the cutoff for poor-quality samples (percent_ribo > 2%). (**E**) Elbow plot heuristic utilized to determine the number of principal components to be used in making the UMAP. Contribution of each principal component levels out between 20 and 30 principal components, and as such 25 principal components were used for the UMAP. (**F**) Clustree diagram representing clustering differences based on resolution. A resolution of 0.6 was chosen based on the relative stability across resolutions while providing a biologically reasonable number of clusters without overly fragmenting clusters.

Nuclei were isolated from the dentate gyrus using the Nuclei EZ prep kit (NUC101; Sigma-Aldrich, St. Louis, MO, USA) as per manufacturer instructions, and each animal was processed individually. Isolated nuclei were submitted for sequencing by the Cincinnati Children’s Hospital and Medical Center Single Cell Genomics Core and were sequenced using the 10x Genomics platform (3’ v3.1 Single Index). Raw reads were aligned to the rat genome (Rnor 6.0) using Cell Ranger (v6.1.2), including both exons and introns. Matrices from Cell Ranger were imported into R (version 4.4.1),, where they were merged. Quality control was conducted using the Seurat (v5.1.0) and SeuratObject (v5.0.2) packages based on established workflows [93,94] (Figure A2). Prior to filtering, 55,863 nuclei were present in our samples. Nuclei were filtered out if they contained less than 500 unique molecular identifiers (UMI), less than 300 features, less 0.8 log10GenesPerUMI, greater than 5% mitochondrial genes, or greater than 2% ribosomal genes. Following filtering, 55,123 nuclei remained for analysis.

The filtered data was then log-normalized with a scaling factor of 10,000. Data was scaled to prevent highly expressed genes from dominating downstream analysis. The first 25 principal components were utilized for dimensional reduction by principal component analysis (PCA) based on the elbow plot leveling out between 20 and 30 principal components (Figure A2E). A UMAP was generated at a resolution of 0.6. A resolution of 0.6 was chosen based on the relative stability across resolutions visualized with a Clustree (Figure A2F) [95] while providing a biologically reasonable number of clusters that were not overly fragmented. Data was integrated using canonical correlation analysis (CCA). CCA identifies conserved sources of variation across groups as a way to identify genes most likely to distinguish cell types [96]. Twenty-four unique clusters were identified, and marker genes for each cluster were identified. The top 30 marker genes for each cluster were imported into the online tool “Annotation of Cell Types” (ACT) [43]. Within ACT, marker genes were compared against a reference database for the mouse hippocampus and the mouse whole brain as no rat-specific reference was available. For several neuronal clusters, inputting the marker genes against the mouse hippocampal and whole-brain datasets resulted in different cluster identification dependent upon the tissue dataset used. In those instances, canonical marker genes were utilized. Cell types containing *Slc17a7* or *Slc17a6*, the genes encoding VGLUT1 and VGLUT2, respectively, and lacking traditional GABAergic markers, *Gabra1*, *Gad1*, and *Gad2*, were labeled as glutamatergic neurons, while clusters with the inverse were labeled as GABAergic neurons. Oligodendrocyte maturity was determined automatically using ACT and represents differences in marker genes associated with the extent of myelination (reviewed in [97]). As such, our “Oligodendrocyte” cluster represents non-myelinating oligodendrocytes, while the “Mature Oligodendrocyte” clusters represent myelinating oligodendrocytes.

Based on our findings from the bulk RNA-seq data, we chose to focus our analysis on microglial and astrocytic clusters. Within these clusters, control (sham or naïve) and TBI animals were compared by differential expression analysis using a non-parametric Wilcoxon Rank Sum test (default in *Seurat*). Differentially expressed genes with an unadjusted *p* < 0.05 were adjusted for multiple testing within GraphPad Prism (version 10.0.3; GraphPad Software Inc., Boston, MA, USA) based on FDR (*Q* = 1%; Benjamini and Hochberg). Genes with *q* < 0.05 with a Log2FC > 1.5 were considered significant DEGs and were visualized as volcano plots using the Enhanced Volcano Package (v1.22.0).

Heatmaps were generated by subsetting the microglial and astrocytic clusters and performing pseudobulking using the AggregateExpression function within Seurat. The top 20 DEGs (*q* < 0.05 and Log2FC > 1.5) based on *p*-value were identified and z-scores were calculated to generate heatmaps using the ComplexHeatmap (v2.20.0) package. Hierarchical clustering was conducted utilizing ComplexHeatmap’s default complete linkage method.

### 4.6. Pathway Analysis

For both the bulk and single-nucleus RNA-seq datasets, we identified signaling pathways associated with our DEGs using iLINCS [98]. The gene names of all DEGs with Log2FC and *p*-values were submitted to iLINCS using the ‘Signatures’ tool. To be more inclusive of DEGs for pathway analysis, all DEGs were passed to iLINCS regardless of Log2FC. As such, iLINCS default absolute Log2FC > 0.5 was used for pathway analysis inclusion. The top 100 recognized DEGs were used as a signature and were then passed to Enrichr [99] within iLINCS. Within Enrichr, we utilized the ‘WikiPathways 2024 Human’ database [100] to identify potential pathways impacted by TBI. Figures were generated within GraphPad Prism by taking the −log (*p*-value) for each pathway. Enrichr calculates *p*-values using a Fisher exact test and adjuste for FDR using Benjamini and Hochberg and a significance of *q* < 0.05 was considered significant.

## Figures and Tables

**Table 1:** Bulk RNA-seq pathway analysis.

Term	DEGs in Dataset/Genes in Pathway	*p*-Value	Adjusted *p*-Value (*q*-Value)	Odds Ratio	Combined Score	DEGs in Pathway
Complement Activation WP545	8/23	9.66 × 10^−14^	2.05 × 10^−11^	120.54	3612.29	*Cfd; C1qb; C1qa; C4a; C1s; C1r; C2; C1qc*
Microglia Pathogen Phagocytosis Pathway WP3937	9/40	2.23 × 10^−13^	2.36 × 10^−11^	66.32	1931.98	*C1qb; C1qq; Tyrobp; Ncf1; Arpc1b; Cyba; Ptpn6; Vav1; C1qc*
Complement System In Neuronal Development And Plasticity WP5090	11/106	3.30 × 10^−12^	2.33 × 10^−10^	26.98	713.36	*C1qb; Cfd; C4a; C1qa; Tgfb; C1s; C1r; Axl; Serping1; C2; C1qc*
Complement And Coagulation Cascades WP558	9/58	8.09 × 10^−12^	4.03 × 10^−10^	41.92	1070.62	*Cfd; C1qb; C1qa; C1s; F10; C1r; Serping1; C2; C1qc*
Dengue 2 Interactions With Complement And Coagulation Cascades WP3896	9/59	9.50 × 10^−12^	4.03 × 10^−10^	41.08	1042.51	*Cfd; C1qb; C1qa; C1s; F10; C1r; Serping1; C2; C1qc*
Oxidative Damage Response WP3941	6/40	3.51 × 10^−8^	1.24 × 10^−6^	38.96	668.74	*C1qb; C1qa; C1s; C1r; C2; C1qc*
Complement System WP2806	7/95	3.68 × 10^−7^	1.12 × 10^−5^	17.71	262.38	*Cfd; C4a; C1s; F10; Serping1; Icam1; C2*
TYROBP Causal Network In Microglia WP3945	6/62	5.17 × 10^−7^	1.37 × 10^−5^	23.63	342.04	*Tyrobp; Npc2; Cd37; Capg; Cxcl16; C1qc*
Allograft Rejection WP2328	6/90	4.70 × 10^−6^	1.11 × 10^−4^	15.73	192.98	*C1qb; C4a; C1qa; Tgfb1; C2; C1qc*
Network Map of SARS-CoV 2 Signaling WP5115	7/254	2.24 × 10^−4^	0.00474164	6.26	52.61	*Lgals3bp; Trpm2; C1s; C1r; Ctsz; Ptpn6; Cxcl16*

Top 10 pathways identified by Enrichr using the WikiPathways 2024 Human dataset. *p*-values computed within Enrichr by Fisher exact test, with *p*-values adjusted for multiple testing using Benjamini–Hochberg method. Odds ratios were derived by Enrichr based on comparisons against computed mean ranks from random gene sets, as analyzed by Fisher exact test. Combined scores were computed by Enrichr from the Fisher exact test by multiplying the −log (*p*-value) by the z-score of the deviation from the expected rank.

**Table 2:** Top 10 differentially expressed genes for microglia clusters.

	Gene Name	*p*-Value	*q*-Value	Avg Log2FC	Pct.1 (Control)	Pct.2 (TBI)
Microglia 1	*Ano5*	1.02 × 10^−12^	1.30 × 10^−10^	−1.52	0.136	0.055
	*Axl*	1.41 × 10^−12^	1.53 × 10^−10^	3.97	0.006	0.065
	*Spata22*	3.94 × 10^−10^	2.73 × 10^−08^	−2.21	0.059	0.013
	*Kmo*	4.18 × 10^−9^	2.28 × 10^−7^	−1.85	0.059	0.016
	*Slfn4*	1.45 × 10^−6^	3.40 × 10^−5^	3.41	0.014	0.054
	*Mx1*	2.38 × 10^−6^	4.97 × 10^−5^	3.28	0.004	0.034
	*Marc1*	3.75 × 10^−6^	7.23 × 10^−5^	4.72	0.001	0.025
	*Tmem154*	4.44 × 10^−6^	8.46 × 10^−5^	−1.51	0.039	0.011
	*LOC100364435*	6.30 × 10^−6^	1.13 × 10^−4^	7.77	0	0.019
	*Gpnmb*	6.69 × 10^−6^	1.19 × 10^−4^	4.27	0.002	0.026
Microglia 2	*Rpl10*	9.75 × 10^−11^	6.39 × 10^−8^	3.95	0	0.018
	*Foxred1*	3.83 × 10^−8^	1.25 × 10^−5^	−1.75	0.213	0.069
	*Cd46*	2.73 × 10^−6^	4.47 × 10^−4^	−1.80	0.186	0.047
	*Ccdc141*	6.03 × 10^−6^	6.58 × 10^−4^	−3.69	0.032	0.002
	*Ccdc88b*	2.44 × 10^−5^	2.02 × 10^−3^	−2.53	0.09	0.013
	*Slfn13*	2.47 × 10^−5^	2.02 × 10^−3^	4.57	0.005	0.101
	*Zfp263*	4.17 × 10^−5^	2.73 × 10^−3^	−6.52	0.037	0
	*Slc2a12*	4.17 × 10^−5^	2.73 × 10^−3^	−6.03	0.037	0
	*Apbb3*	4.40 × 10^−5^	2.74 × 10^−3^	−1.88	0.16	0.04
	*Fbxw9*	1.06 × 10^−4^	4.78 × 10^−3^	−1.98	0.106	0.025

Top 10 DEGs for microglial clusters. A complete list of DEGs can be found in the Supplementary Materials.

**Table 3:** Pathway analysis for microglial clusters for single-nucleus RNA-seq.

	Term	DEGs in Dataset/Genes in Pathway	*p*-Value	Adjusted *p*-Value (*q*-Value)	Odds Ratio	Combined Score	DEGs in Pathway
Microglia 1	Complement System In Neuronal Development And Plasticity WP5090	7/106	1.03 × 10^−6^	2.07 × 10^−4^	15.05	207.58	*C1qb; C1qa; Axl; Mbp; Itgav; Mertk; C1qc*
	Neuroinflammation And Glutamatergic Signaling WP5083	7/140	6.59 × 10^−6^	6.66 × 10^−4^	11.19	133.45	*Camk2b; Gria2; Camk2D; Slc17a7; Disc1; Grm1; Gls*
	Disruption Of Postsynaptic Signaling By CNV WP4875	4/33	2.15 × 10^−5^	1.45 × 10^−3^	28.55	306.79	*Camk2b; Camk2d; Nrxn3; Grm1*
	Microglia Pathogen Phagocytosis Pathway WP3937	4/40	4.68 × 10^−5^	2.36 × 10^−3^	22.99	229.20	*C1qb; Vav3; C1qa; C1qc*
	Serotonin And Anxiety WP3947	3/17	7.84 × 10^−5^	3.17 × 10^−3^	43.93	415.32	*Camk2b; Ppp3ca; Grm1*
	Complement Activation WP545	3/23	2.00 × 10^−4^	6.72 × 10^−3^	30.74	261.88	*C1qb; C1qa; C1qc*
	Photodynamic Therapy-Induced Unfolded Protein Response WP3613	3/27	3.25 × 10^−4^	8.30 × 10^−3^	25.61	205.71	*Hspa5; Calr; Hsp90b1*
	Spinal Cord Injury WP2431	5/119	3.29 × 10^−4^	8.30 × 10^−3^	9.13	73.26	*C1qb; Fkbp1a; Ppp3ca; Ptpra; Mbp*
	Primary Focal Segmental Glomerulosclerosis FSGS WP2572	4/72	4.66 × 10^−4^	1.05 × 10^−2^	12.15	93.21	*Camk2b; Trpc6; Ctsl; Itgav*
	Cell Lineage Map For Neuronal Differentiation WP5417	5/132	5.29 × 10^−4^	1.07 × 10^−2^	8.19	61.82	*Map2; Mbp; Slc17a7; S100b; Gls*
Microglia 2	Genes Controlling Nephrogenesis WP4823	4/44	6.85 × 10^−5^	1.10 × 10^−2^	20.69	198.37	*Robo2; Notch2; Gli3; Vegfa*
	Osteoarthritic Chondrocyte Hypertrophy WP5373	3/50	2.00 × 10^−3^	1.11 × 10^−1^	13.06	81.16	*Jund; Junb; Vegfa*
	11P11 2 Copy Number Variation Syndrome WP5348	3/56	2.77 × 10^−3^	1.11 × 10^−1^	11.58	68.18	*B4gat1; Nrxn1; Gli3*
	Oncostatin M Signaling WP2374	3/56	2.77 × 10^−3^	1.11 × 10^−1^	11.58	68.18	*Jund; Junb; Vegfa*
	Hypertrophy Model WP516	2/20	4.43 × 10^−3^	1.42 × 10^−1^	22.54	122.14	*Jund; Vegfa*
	Axon Guidance WP5289	3/72	5.64 × 10^−3^	1.45 × 10^−1^	8.89	46.03	*Robo2; Robo3; Lrrc4c*
	Hippocampal Synaptogenesis And Neurogenesis WP5231	2/24	6.36 × 10^−3^	1.45 × 10^−1^	18.44	93.27	*Nrxn1; Ncam1*
	EGF EGFR Signaling WP437	4/159	8.36 × 10^−3^	1.67 × 10^−1^	5.31	25.40	*Ptprr; Jund; Inpp5d; Ncoa3*
	GDNF RET Signaling Axis WP4830	2/30	9.83 × 10^−3^	1.75 × 10^−1^	14.48	66.95	*Robo2; Gli3*
	Disruption of Postsynaptic Signaling by CNV WP4875	2/33	1.18 × 10^−2^	1.89 × 10^−1^	13.08	58.06	*Nrxn1; Dlgap1*

Pathway analysis from microglial clusters for single-nucleus RNA-seq: For Microglia 1 and Microglia 2 clusters, the top 10 pathways identified by Enrichr using the WikiPathways 2024 Human dataset. *p*-values computed within Enrichr by Fisher exact test, with *p*-values adjusted for multiple testing using Benjamini–Hochberg method. Odds ratios were derived by Enrichr based on comparisons against computed mean ranks from random gene sets, as analyzed by Fisher exact test. Combined scores were computed by Enrichr from the Fisher exact test by multiplying the −log (*p*-value) by the z-score of the deviation from the expected rank.

**Table 4:** Top 10 differentially expressed genes for astrocyte clusters.

	Gene Name	*p*-Value	*q*-Value	Avg Log2FC	Pct.1 (Control)	Pct.2 (TBI)
Astrocyte 1	*Pld5*	1.29 × 10^−54^	3.51 × 10^−51^	3.18	0.02	0.157
	*Nme4*	1.32 × 10^−29^	7.18 × 10^−27^	8.73	0	0.044
	*Plk5*	2.35 × 10^−24^	5.81 × 10^−22^	1.60	0.043	0.148
	*Celsr3*	6.06 × 10^−20^	5.89 × 10^−18^	1.52	0.025	0.098
	*LOC100364435*	1.22 × 10^−17^	7.04 × 10^−16^	8.36	0	0.02
	*Dlk2*	1.32 × 10^−17^	7.48 × 10^−16^	1.52	0.026	0.091
	*Rps23*	2.04 × 10^−17^	1.12 × 10^−15^	3.61	0.004	0.087
	*Asb15*	1.18 × 10^−15^	4.04 × 10^−14^	4.28	0.001	0.026
	*Rpl18a*	1.09 × 10^−13^	2.39 × 10^−12^	9.41	0	0.063
	*Epha8*	2.69 × 10^−13^	5.5 × 10^−12^	1.64	0.027	0.084
Astrocyte 2	*AABR07000398.1*	4.15 × 10^−64^	4.61 × 10^−61^	1.55	0.624	0.824
	*Map1a*	4.42 × 10^−45^	3.28 × 10^−42^	1.83	0.26	0.551
	*S100b*	1.82 × 10^−36^	8.09 × 10^−34^	1.55	0.235	0.486
	*Hpcal4*	2.49 × 10^−20^	4.26 × 10^−18^	1.81	0.142	0.316
	*Agap2*	3.42 × 10^−20^	5.43 × 10^−18^	1.71	0.158	0.332
	*Ddn*	4.92 × 10^−18^	5.76 × 10^−16^	1.69	0.1	0.247
	*Mapk8ip2*	2.06 × 10^−16^	1.64 × 10^−14^	1.51	0.116	0.265
	*Ubc*	1.36 × 10^−14^	8.40 × 10^−13^	1.55	0.1	0.23
	*Rpl10*	5.95 × 10^−12^	2.50 × 10^−10^	1.58	0.005	0.028
	*Gnas.1*	1.92 × 10^−11^	7.11 × 10^−10^	1.62	0.068	0.166
Astrocyte 3	*Pld5*	2.25 × 10^−30^	7.50 × 10^−28^	2.93	0.051	0.256
	*Rpl10*	1.16 × 10^−22^	1.78 × 10^−20^	7.65	0	0.106
	*Slc9b1*	2.17 × 10^−17^	1.38 × 10^−15^	−1.71	0.244	0.085
	*Jhy*	1.16 × 10^−15^	5.95 × 10^−14^	−1.63	0.237	0.083
	*Begain*	1.67 × 10^−13^	5.96 × 10^−12^	1.85	0.101	0.257
	*Nme4*	1.91 × 10^−13^	6.76 × 10^−12^	6.14	0	0.049
	*LOC100364435*	6.10 × 10^−13^	1.98 × 10^−11^	5.89	0	0.022
	*Rapgefl1*	6.24 × 10^−12^	1.63 × 10^−10^	1.73	0.112	0.264
	*Hba-a2*	3.53 × 10^−11^	7.71 × 10^−10^	−4.42	0.077	0.006
	*Cacna1h*	5.23 × 10^−11^	1.10 × 10^−9^	1.50	0.102	0.239

Top 10 DEGs for astrocyte clusters. A complete list of DEGs can be found in the Supplementary Materials.

**Table 5:** Pathway analysis of astrocyte clusters for single-nucleus RNA-seq.

	Term	DEGs in Dataset/Genes in Pathway	*p*-Value	Adjusted *p*-Value (*q*-Value)	Odds Ratio	Combined Score	DEGs in Pathway
Astrocyte 1	Neuroinflammation And Glutamatergic Signaling WP5083	9/140	3.45 × 10^−8^	5.08 × 10^−6^	14.93	256.43	*Camk2b; Camk2a; Slc1a2; Slc1a3; Nsmf; Slc17a7; Il6r; Camkk1; Grin1*
	Fragile X Syndrome WP4549	7/122	2.65 × 10^−6^	1.94 × 10^−4^	12.95	166.31	*Camk2b; Map1b; Camk2a; Agap2; Dlgap3; Dnm; Grin1*
	Cell Lineage Map For Neuronal Differentiation WP5417	7/132	4.47 × 10^−6^	2.13 × 10^−4^	11.91	146.68	*Slc1a2; Rimbp2; Slc1a3; Slc17a7; Bsn; Cacng2; Grin1*
	Hippocampal Synaptogenesis And Neurogenesis WP5231	4/24	5.79 × 10^−6^	2.13 × 10^−4^	41.42	499.47	*Camk2b; Nrxn1; Camk2a; Camkk1*
	Disruption Of Postsynaptic Signaling By CNV WP4875	4/33	2.15 × 10^−5^	6.33 × 10^−4^	28.55	306.79	*Camk2b; Nrxn1; Camk2a; Grin1*
	NO cGMP PKG Mediated Neuroprotection WP4008	4/46	8.17 × 10^−5^	2.00 × 10^−3^	19.70	185.43	*Camk2b; Camk2a; Pde2a; Grin1*
	Microtubule Cytoskeleton Regulation WP2038	4/48	9.67 × 10^−5^	2.03 × 10^−3^	18.80	173.82	*Tiam1; Ntrk3; Map1b; Ephb2*
	Synaptic Vesicle Pathway WP2267	3/51	2.12 × 10^−3^	3.90 × 10^−2^	12.79	78.73	*Slc1a3; Slc17a7; Dnm1*
	Phosphodiesterases In Neuronal Function WP4222	3/54	2.50 × 10^−3^	4.08 × 10^−2^	12.04	72.12	*Camk2a; Pde2A; Grin1*
	NAD Metabolism WP3644	2/16	2.84 × 10^−3^	4.17 × 10^−2^	28.99	170.01	*Nt5e; Nmnat2*
Astrocyte 2	Disruption Of Postsynaptic Signaling By CNV WP4875	6/33	1.33 × 10^−8^	2.37 × 10^−6^	46.98	851.93	*Camk2b; Nlgn1; Dlg2; Nrxn1; Nrxn3; Dlgap1*
	Neuroinflammation And Glutamatergic Signaling WP5083	8/140	5.10 × 10^−7^	4.54 × 10^−5^	13.02	188.67	*Camk2b; Grm5; Cfl1; Nsmf; Calm1; Adcy8; Glul; Gfap*
	Cell Lineage Map For Neuronal Differentiation WP5417	7/132	4.47 × 10^−6^	2.65 × 10^−4^	11.91	146.68	*Nlgn1; Dlg2; Aqp4; Mbp; S100b; Glul; Gfap*
	ADHD And Autism ASD Pathways WP5420	10/370	1.64 × 10^−5^	7.31 × 10^−4^	6.03	66.44	*Gabrb1; Grm5; Nlgn1; Dlg2; Syt1; Nrxn1; Nrxn3; Dlgap1; Calm1; Glul*
	Common Pathways Underlying Drug Addiction WP2636	4/41	5.17 × 10^−5^	1.84 × 10^−3^	22.37	220.78	*Grm5; Adcy8; Calm1; Actb*
	Calcium Regulation In Cardiac Cells WP536	6/151	1.11 × 10^−4^	3.31 × 10^−3^	8.70	79.15	*Camk2b; Ywhab; Calm1; Adcy8; Calm2; Kcnj3*
	Myometrial Relaxation And Contraction Pathways WP289	6/156	1.33 × 10^−4^	3.39 × 10^−3^	8.40	74.99	*Camk2b; Ywhab; Calm1; Adcy8; Calm2; Actb*
	Hippocampal Synaptogenesis And Neurogenesis WP5231	3/24	2.27 × 10^−4^	5.06 × 10^−3^	29.28	245.59	*Camk2b; Nrxn1; Calm1*
	Glial Cell Differentiation WP2276	2/7	5.11 × 10^−4^	1.01 × 10^−2^	81.20	615.40	*Plb1; Mbp*
	Sudden Infant Death Syndrome SIDS Susceptibility Pathways WP706	5/157	1.16 × 10^−3^	2.06 × 10^−2^	6.84	46.24	*Ywhab; Plp1; Aqp4; Aldoa; Sptbn1*
Astrocyte 3	Fragile X Syndrome WP4549	10/122	5.35 × 10^−10^	7.70 × 10^−8^	19.63	419.10	*Cyfip2; Gria1; Camk2b; Ppp3ca; Grin2a; Map1b; Camk2a; Agap2; Dnm1; Grin1*
	Disruption Of Postsynaptic Signaling By CNV WP4875	6/33	1.33 × 10^−8^	9.60 × 10^−7^	46.98	851.93	*Camk2b; Ryr2; Grin2a; Nrxn1; Camk2a; Grin1*
	Synaptic Vesicle Pathway WP2267	6/51	2.02 × 10^−7^	9.67 × 10^−6^	28.16	434.20	*Snap25; Slc1a3; Atp1a2; Slc17a7; Cplx2; Dnm1*
	Neuroinflammation And Glutamatergic Signaling WP5083	8/140	5.10 × 10^−7^	1.84 × 10^−5^	13.02	188.67	*Gria1; Camk2b; Grin2a; Camk2a; Slc1a2; Slc1a3; Slc17a7; Grin1*
	NRXN1 Deletion Syndrome WP5398	4/17	1.33 × 10^−6^	3.84 × 10^−5^	63.74	862.34	*Gria1; Grin2a; Nrxn1; Grin1*
	Cell Lineage Map For Neuronal Differentiation WP5417	7/132	4.47 × 10−^6^	1.07 × 10^−4^	11.91	146.68	*Snap25; Slc1a2; Rimbp2; Slc1a3; Slc17a7; Bsn; Grin1*
	Phosphodiesterases In Neuronal Function WP4222	5/54	7.36 × 10^−6^	1.51 × 10^−4^	21.32	252.03	*Gria1; Pde11A; Grin2A; Camk2a; Grin1*
	Common Pathways Underlying Drug Addiction WP2636	4/41	5.17 × 10^−5^	9.30 × 10^−4^	22.37	220.78	*Gria1; Grin2a; Camk2a; Grin1*
	NO cGMP PKG Mediated Neuroprotection WP4008	4/46	8.17 × 10^−5^	1.31 × 10^−3^	19.70	185.43	*Camk2b; Grin2A; Camk2a; Grin1*
	Hippocampal Synaptogenesis And Neurogenesis WP5231	3/24	2.27 × 10^−4^	3.28 × 10^−3^	29.28	245.59	*CAamk2b; Nrxn1; Camk2a*

Pathway analysis from astrocyte clusters for single-nucleus RNA-seq: for Astrocyte 1, Astrocyte 2, and Astrocyte 3 clusters, the top 10 pathways identified by Enrichr using the WikiPathways 2024 Human dataset. *p*-values computed within Enrichr by Fisher exact test, with *p*-values adjusted for multiple testing using Benjamini–Hochberg method. Odds ratios were derived by Enrichr based on comparisons against computed mean ranks from random gene sets, as analyzed by Fisher exact test. Combined scores were computed by Enrichr from the Fisher exact test by multiplying the −log (*p*-value) by the z-score of the deviation from the expected rank.

## Data Availability

Raw data and processed data are available on Gene Expression Omnibus (GSE307917), and the Cell Ranger script and all relevant R-code are available at https://github.com/NgwenyaLab/DeSana_and_Alfawares_2025 (accessed on 30 July 2025).

## Supplementary Materials

The following supporting information can be downloaded at https://www.mdpi.com/article/10.3390/ijms26189140/s1.

## Abbreviations

The following abbreviations are used in this manuscript:

TBI	Traumatic Brain Injury
LFPI	Lateral Fluid Percussion Injury
RRT	Righting Reflex Time
UMAP	Uniform Manifold Approximation and Projection
DEG	Differentially Expressed Gene
ACT	Annotation of Cell Type
MAC	Membrane Attack Complex
fB	Factor B
sCR1	Soluble CR1
sCrry	Soluble Complement Inhibitor
PBS	Phosphate-Buffered Saline
logCPM	Log2 Counts Per Million
log2FC	Log2 Fold Change
UMI	Unique Molecular Identifier
PCA	Principal Component Analysis
CCA	Canonical Correlation Analysis
Stdev	Standard Deviation
RNA-seq	Ribonucleic Acid Sequencing

## Appendix A

**Table A1:** Nuclei counts per cluster for each injury condition.

Cluster Name	Nuclei Counts (Control)	Nuclei Counts (TBI)	Control % of Total	TBI % of Total
Astrocyte 1	2307	1308	8.18%	4.86%
Astrocyte 2	753	1144	2.67%	4.25%
Astrocyte 3	743	696	2.63%	2.59%
Endothelial Cell	197	237	0.70%	0.88%
GABAergic Neuron 1	623	702	2.21%	2.61%
GABAergic Neuron 2	531	681	1.88%	2.53%
GABAergic Neuron 3	460	699	1.63%	2.60%
Glutamatergic Neuron 1	6610	5348	23.43%	19.88%
Glutamatergic Neuron 2	3936	3096	13.95%	11.51%
Glutamatergic Neuron 3	3082	2220	10.92%	8.25%
Glutamatergic Neuron 4	2949	2327	10.45%	8.65%
Glutamatergic Neuron 5	902	996	3.20%	3.70%
Glutamatergic Neuron 6	672	662	2.38%	2.46%
Glutamatergic Neuron 7	419	377	1.48%	1.40%
Glutamatergic Neuron 8	342	442	1.21%	1.64%
Mature Oligodendrocyte 1	848	1667	3.01%	6.20%
Mature Oligodendrocyte 2	263	458	0.93%	1.70%
Mature Oligodendrocyte 3	19	30	0.07%	0.11%
Microglia 1	900	1611	3.19%	5.99%
Microglia 2	188	447	0.67%	1.66%
Oligodendrocyte	155	194	0.55%	0.72%
Oligodendrocyte Precursor 1	995	1128	3.53%	4.19%
Oligodendrocyte Precursor 2	251	369	0.89%	1.37%
Pericyte	71	68	0.25%	0.25%

Nuclei counts per cluster by injury condition: Table contains the number of nuclei present in each cluster on the UMAP for control and TBI animals. The two right-most columns contain the number of nuclei in a given cluster divided by the total number of nuclei for each injury condition, represented as a percentage.
